
JOURNAL INFORMATION
==============================
NLM Title Abbreviation: Atlanta Med Surg J (1884)
NLM Journal ID: 101251623

Atlanta Medical and Surgical Journal (1884)

Publisher: Biomedical Journal Digitization Project

ARTICLE INFORMATION
==============================
PMCID: PMC9201861
PMID: 35829609
Article version: 1
Subjects: Editorial Notes and Comments

Ear Disease and Life Insurance

Print publication date: 1899 Feb
Volume: 15
Volume Series: New Ser
Issue: 12
First page: 822
Last page: 824
License: This work is free of known copyright restrictions. For more information, please see PMC Back Issue Digitization.
License URL: https://creativecommons.org/publicdomain/mark/1.0/

==============================

### EDITORIAL NOTES AND COMMENTS.

The Business office of THE JOURNAL is 305, 306 Fitten Building.

The Editorial office is Rooms 401, 402 Grand Opera House.

Address all Business communications to Dr. M. B. Hutchins, Mgr.

Make remittances payable to THE ATLANTA MEDICAL AND SURGICAL JOURNAL.

On matters pertaining to the Editorial and Original communications address Dr. Dunbar Roy, Grand Opera House, Atlanta.

Reprints of original articles will be furnished at cost price. Requests for the same should always be made *on the manuscript*.

We will present, post-paid, on request, to each contributor of an original article, twenty (20) marked copies of THE JOURNAL containing such article.

### EAR DISEASE AND LIFE INSURANCE.

THIS subject has recently been handled by Dr. P. M. McBride, of Edinburgh, in an article read before the British Medical Association. The subject is a timely one and has been very rarely considered in its relationship to life insurance. We cannot fully agree with Dr. McBride in many of his deductions, but he has certainly presented much food for thought. We believe that he is too extreme in his views in rejecting persons for insurance who show certain lesions of the ear. For instance we find these words, "thus I would certainly reject a case in which polypi or granulations were present." Such a statement as that could not be allowed to go unchallenged. After all, Dr. McBride speaks from his own personal experience, and perhaps such a pathological condition may be always serious in Edinburgh, but this certainly does not apply to this section of the country. The presence of polypi or granulations in the middle ear does not indicate that the ear itself is destroyed much less that it is likely to prove fatal to life. Ears of this nature are always a menace to their possessor and yet we have seen such a condition cured time and again and a useful aural appendage restored. We do not think that such a sweeping statement should be made, but rather hold such an applicant in abeyance until it is seen whether or not the pathological condition can be removed. Many excellent risks would otherwise be debarred from active life insurance should such ideas be adhered to.

Then again we read the following statement from the same article: "The presence of diseased bone is of course an absolute bar to acceptance, while the same is true of cholesteatoma of the middle ear." This, too, should be qualified, for there are cases of suppurative otitis media which can be cured, and these same cases may at the beginning show some diseased bone. Here again an applicant should not be rejected until it is seen whether or not such an ear is curable. No broad absolute statement like the above should be made. In concluding, Dr. McBride sums up certain points well worthy to be known:

"There are, however, a considerable number of cases in which none of these complications exist—in fact, where we have only perforated membrane through which pus is discharged. In estimating such cases we must be guided by:

"1. *The Perforation.*—If it be small and high up in the membrane the risks are greater than if it be large and lower down.

"2. *The Amount and Character of the Discharge.*—If this be in very large quantity, and more particularly if it be copious and fetid, the case should be rejected.

"3. *The Social Position and Habits of the Patient.*—I cannot but lay very great stress upon this point, for in my experience fatal cases of ear disease are relatively far more common among the poorer classes, in whom there is generally a tendency to neglect a discharging ear so long as no painful symptoms arise.

"Given a case of uncomplicated ear suppuration, what are the objective characteristics which make us take a relatively favorable view of the case?

"In the first place the discharge must be small in amount and not fetid. A source of fallacy must be guarded against here. We sometimes meet with very large perforations amounting to destruction of almost the whole tympanic membrane, in which there is only a small amount of pus secreted, and no very marked fetor is detected by the superficial observer. In these cases, however, a bent probe introduced into the attic of the tympanum will be found extremely offensive when removed. Again, in some cases of perforation of Shrapnel's membrane the discharge may for a time be almost sweet; but, again, a probe introduced can be made to reveal the fallacy. There is, however, another form of middle-ear suppuration, in which the perforation is of a fair size and usually in the lower part of the membrane, the discharge is slightly, if at all, fetid, and may be muco-purulent in character, and often ceases for long periods after appropriate treatment. In these cases, I think we may say that there is a minimum of risk. Again, when a discharge has lasted for many years without ever causing pain, when it has remained small in amount, and when, although untreated, it has not given rise to granulations, the conditions seem to me relatively favorable."

"I have not attempted to give a complete account of ear disease in its relation to life insurance, but rather to indicate some of the points which seem to me deserving of consideration. I leave it to those who are statisticians to decide how far the figures which I have adduced, meager as they are, can be utilized to arrive at any definite conclusion as to the dangers of suppurative ear disease, more especially when they are considered together with the undoubted fact that some forms are very much less likely than others to lead to dangerous complications."
